# A modified Michaelis-Menten equation estimates growth from birth to 3 years in healthy babies in the USA

**DOI:** 10.1186/s12874-024-02145-1

**Published:** 2024-02-01

**Authors:** William A. Walters, Catherine Ley, Trevor Hastie, Ruth E. Ley, Julie Parsonnet

**Affiliations:** 1 Department of Microbiome Science, Max Planck Institute for Biology, Tübingen, Germany; 2 Division of Infectious Diseases and Geographic Medicine, Department of Medicine, Stanford University School of Medicine, 300 Pasteur Drive, Stanford, CA 94305-5170 USA; 3 Departments of Statistics and of Biomedical Data Sciences, Stanford University, Stanford, CA USA; 4 Department of Epidemiology and Population Health, Stanford University School of Medicine, Stanford, CA USA

**Keywords:** Michaelis–Menten equation, Growth, Birth cohort, Height estimation, Weight estimation

## Abstract

**Background:**

Standard pediatric growth curves cannot be used to impute missing height or weight measurements in individual children. The Michaelis–Menten equation, used for characterizing substrate-enzyme saturation curves, has been shown to model growth in many organisms including nonhuman vertebrates. We investigated whether this equation could be used to interpolate missing growth data in children in the first three years of life and compared this interpolation to several common interpolation methods and pediatric growth models.

**Methods:**

We developed a modified Michaelis–Menten equation and compared expected to actual growth, first in a local birth cohort (*N* = 97) then in a large, outpatient, pediatric sample (*N* = 14,695).

**Results:**

The modified Michaelis–Menten equation showed excellent fit for both infant weight (median RMSE: boys: 0.22 kg [IQR:0.19; 90% < 0.43]; girls: 0.20 kg [IQR:0.17; 90% < 0.39]) and height (median RMSE: boys: 0.93 cm [IQR:0.53; 90% < 1.0]; girls: 0.91 cm [IQR:0.50;90% < 1.0]). Growth data were modeled accurately with as few as four values from routine well-baby visits in year 1 and seven values in years 1–3; birth weight or length was essential for best fit. Interpolation with this equation had comparable (for weight) or lower (for height) mean RMSE compared to the best performing alternative models.

**Conclusions:**

A modified Michaelis–Menten equation accurately describes growth in healthy babies aged 0–36 months, allowing interpolation of missing weight and height values in individual longitudinal measurement series. The growth pattern in healthy babies in resource-rich environments mirrors an enzymatic saturation curve.

**Supplementary Information:**

The online version contains supplementary material available at 10.1186/s12874-024-02145-1.

## Background

Height, weight, and growth are foundational indicators of child health. Growth charts, created by the World Health Organization [1] and the US Centers for Disease Control and Prevention [2], serve as clinical references to evaluate individual pediatric physical sizes and growth rates against population means. These reference ranges represent cross-sectional information from tens to tens of thousands of children per age group. Longitudinal studies, however, demonstrate the unpredictability of individual patterns, with short growth spurts punctuating periods of minimal growth (i.e., a saltatory pattern) [3, 4]. Thus, actual growth for an individual child is statistically unique [5].

Stanford’s Outcome Research Kids (STORK) is a birth cohort recruited in the San Francisco Bay Area, California, designed to evaluate the impact of infections on growth from birth to age 36 months [6]. In this project, some infants were missing necessary time-specific weight measurements. We sought to identify an empirical longitudinal growth model that would provide the best interpolation of missing weight values given only the available weight values for that individual baby—in essence, a function that would smooth noisy existent data to fit a line and that was simple, to avoid overfitting.

The Michaelis–Menten equation was originally used in biochemistry to describe how substrate concentration affects the rate of enzyme catalysis [7]. The equation was subsequently slightly modified and applied to a wide range of chemical and biological processes, ranging from antibody development to soil microbial activity to tree growth [8–10]. The Michaelis–Menten equation also describes growth accurately in fish, birds and mammals of various sizes [11]. To date, however, the equation has not been used to model human growth.

We applied a modified Michaelis–Menten equation to each STORK baby’s individual weight curve and evaluated its fit. We then validated the use of this equation for weight and also height using a large longitudinal dataset from healthy babies (Stanford Medicine Research Data Repository (STARR)) and additionally identified those well-baby visit timepoint combinations essential for best model fit. We evaluated the accuracy of this equation to predict weight and height during the second and/or third year of life when using growth measures from earlier timepoints. Finally, we compared interpolation as performed by the modified Michaelis–Menten equation to that of several commonly used interpolation methods and pediatric growth models.

## Methods

### Babies

Detailed methods for the STORK birth cohort have been described previously [6]. In brief, a multiethnic cohort of mothers and babies was followed from the second trimester of pregnancy to the babies’ third birthday. Healthy women aged 18–42 years with a single-fetus pregnancy were enrolled. Households were visited every four months until the baby’s third birthday (nine baby visits), with the weight of the baby at each visit recorded in pounds. Medical charts were abstracted for birth weight and length. All data were managed in REDCap [12] hosted at Stanford University.

STARR (starr.stanford.edu) contains electronic medical record information from all pediatric and adult patients seen at Stanford Health Care (Stanford, CA). STARR staff provided anonymized information (weight, height and age in days for each visit through age three years; sex; race/ethnicity) for all babies during the period 03/2013–01/2022 followed from birth to at least 36 months of age with at least five well-baby care visits over the first year of life.

### Statistical analysis

All observed weight and height values were evaluated in kilograms (kg) and centimeters (cm), respectively. Any values assessed beyond 1,125 days (roughly 36 months) and values for height and weight deemed implausible by at least two reviewers (e.g., significant losses in height, or marked outliers for weight and height) were excluded from the analysis. Additionally, weights assessed between birth and 19 days were excluded, as weight loss often occurs immediately after birth, and approximately 95% of babies return to their birth weight by 19 days [13]. At least five observations across the 36-month period were required: babies with fewer than five weight or height values after the previous criteria were excluded from analyses.

#### Model

We developed our weight model using values from STORK babies and then replicated it with values from the STARR babies. Height models were evaluated in STARR babies only because STORK data on height were scant.

The Michaelis-Menten equation is described as follows:

$${\text{v}}={{\text{V}}}_{{\text{max}}}\left(\left[{\text{S}}\right]/{{\text{K}}}_{{\text{m}}}+\left[{\text{S}}\right])\right)$$

where v is the rate of product formation, V_max_ is the maximum rate of the system, [S] is the substrate concentration, and K_m_ is a constant based upon the enzyme’s affinity for the particular substrate.

For this study the equation became:

$$P=\text{a}1\left(\textit{Age}/\left(\text{b}1+A\textit{g}e\right)\right)+\text{c}1$$

where *P* was the predicted value of weight (kg) or height (cm), *Age* was the age of the infant in days, and c1 was an additional constant over the original Michaelis–Menten equation that accounted for the infant’s non-zero weight or length at birth. Each of the parameters a1, b1 and c1 was unique to each child and was calculated using the nonlinear least squares (nls) method. In our case, weight data were fitted to a model using the statistical language R (version 3.4.0) [14], by calling the formula nls() with the following parameters:

$${\text{fitted}}\_\mathrm{model }<-{\text{nls}}({\text{weights}}\sim ({\text{c}}1+({\text{a}}1*{\text{ages}})/({\text{b}}1+{\text{ages}})),\mathrm{ start }=\mathrm{ list}({\text{a}}1 = 5,\mathrm{ b}1 = 20,\mathrm{ c}1=2.5))$$

where weights and ages were vectors of each subject’s weight in kg and age in days. The default Gauss–Newton algorithm was used. The optimization objective is not convex in the parameters, and can suffer from local optima and boundary conditions. In such cases good starting values are essential: the starting parameter values (a1 = 5, b1 = 20, c1 = 2.5) were adjusted manually (based upon repeated trials with a range of values) using the STORK dataset to minimize model failures; these tended to occur when the parameter values, particularly a1 and b1, increased without bound during the iterative steps required to optimize the model. Using higher starting a1 and b1 parameter values, i.e., closer to the mean/median values upon which the nls function previously converged, gave similar a1 and b1 parameter values, but also a higher rate of model failures due to more a1 and b1 values increasing without bound. These same parameter values were used for the larger STARR dataset.

The starting height parameter values for height modeling were higher than those for weight modeling, due to the different units involved (cm vs. kg) (a1 = 60, b1 = 530, c1 = 50). Correlations between the c1 parameter and birth weight or birth length for all babies by sex and by study were evaluated using Spearman’s rank coefficient.

Because this was a non-linear model, goodness of fit was assessed primarily via root mean squared error (RMSE) for both weight and height [15]. The values of RMSE are in the same units as those measured (kg or cm) and can be used as estimates of the deviation in values predicted by the model from the observed values (lower RMSE values indicate better model fit). To evaluate the effect of age on the RMSE, we considered the RMSE for each timepoint and visualized all RMSE vs. age.

#### Imputation tests

To test for the influence of specific time points on the models, we limited our analysis to STARR babies with all recommended well-baby visits (12 over three years [16]). Each scheduled visit except day 1 occurred in a time window around the expected well-baby visit (Visit1: Day 1, Visit2: days 20–44, Visit3: 46–90, Visit4: 95–148, Visit5: 158–225, Visit6: 250–298, Visit7: 310–399, Visit8: 410–490, Visit9: 500–600, Visit10: 640–800, Visit11: 842–982, Visit12: 1024–1125). We considered two different sets: infants with all scheduled visits in the first year of life (seven total visits) and those with all scheduled visits over the full three-year timeframe (12 total visits). We fit these two sets to the model, identifying baseline RMSE. Then, every visit, and every combination of two to five visits were dropped, so that the RMSE or model failures for combination of visits could be compared to baseline.

#### Prediction

We sought to predict weight or height at 36 months (Y3) from growth measures assessed only up to 12 months (Y1) or to 24 months (Y1 + Y2), utilizing the “last value” approach [17]. In brief, the last observation for each child (here, growth measures at 36 months) is used to assess overall model fit, by focusing on how accurately the model can extrapolate the measure at this time point. We identified all STARR infants with at least five time points in Y1 and at least two time points in both Y2 and Y3, with the selection of these time points based on maximizing the number of later time points within the constraints of the well-baby visit schedule for Y2 and Y3. The per-subject set of time points (Y1-Y3) was fitted using the modified Michaelis–Menten equation and the mean squared error was calculated, acting as the “baseline” error. The model was then run on the subset of Y1 only and of Y1 + Y2 only. To test predictive accuracy of these subsets, the RMSE was calculated using the actual weights or heights versus the predicted weights or heights of the three time series.

#### Comparison with other models

We examined how well the modified Michaelis–Menten equation performed interpolation in STARR babies compared to ten other commonly used interpolation methods and pediatric growth models including: (1) the ‘last observation carried forward’ model; (2) the linear model; (3) the robust linear model (RLM method, base R MASS package); (4) the Laird and Ware linear model (LWMOD method) [18]; (5) the generalized additive model (GAM method) [19]; (6) locally estimated scatterplot smoothing (LOESS method, base R stats package); (7) the smooth spline model (smooth.spline method, base R stats package); (8) the multilevel spline model (Wand method) [20]; (9) the SITAR (superimposition by translation and rotation) model [21] and (10) fast covariance estimation (FACE method) [22].

Model fit used the holdout approach [17]: a single datapoint (other than birth weight or birth length) was randomly removed from each subject, and the RMSE of the removed datapoint was calculated as the model fitted to the remaining data.

The hbgd package [17] was used to fit all models except the ‘last observation carried forward’ model, the linear model and the SITAR model. For the ‘last observation carried forward’ model, the holdout data point was interpolated by the last observation by converting the random holdout value to NA and then using the function na.locf() from the zoo R package [23]. For the simple linear model, the holdout-filtered data were used to determine the slope and intercept via R’s lm() function, which were then used to calculate the holdout value. For the SITAR model, each subject was fitted calling the sitar() function with df = 2 to minimize failures, and the RMSE of the random holdout point was subsequently calculated with the predict() function. For this analysis, set.seed(1234) was used to initialize the pseudorandom generator.

All analyses were performed in R^14^ (3.4.0 for the modified Michaelis–Menten equation fitting, 4.1.3 for holdout testing; R configuration data, scripts and study data available at  10.5061/dryad.4j0zpc8jf). An R script to run the modified Michaelis–Menten equation can be downloaded at: https://gist.github.com/walterst/ede8b883d4f9acaf45ec9e2b0ec811fe.

## Results

A total of 126 STORK and 14,817 STARR babies were considered for this analysis (Supplemental Fig. 1). After excluding values per protocol, 97 (77.0%) STORK and 14,695 (99.2%) STARR babies had sufficient measurements to be included in the weight analyses. For height, examined only in STARR, 11,655 (78.7%) babies were included.

The sex of infants was similar in both cohorts but STORK babies were slightly heavier than STARR babies (Table 1). For STORK babies, weight values were spread fairly consistently across the 36 months by design; for STARR babies, the number of weight and height timepoints per subject was variable (range: weight: 5–15; height: 5–13).

**Table 1:** Characteristics of STORK and STARR babies

		**STORK**		**STARR**	
		**N**	**Statistic** ^a^	**N**	**Statistic**
Babies in weight analyses		97		14,695	
Babies in height analyses		NA^b^		11,655	
Female		49	50.5	7162	48.7
Birthweight	kg	96	3.42 (0.46)	14,695	3.28 (0.50)
Birth length	cm	NA		11,655	50.23 (2.58)
Weight at ~ 36 months^c^	kg	35	15.48 (2.76)	3,117	14.72 (1.84)
Height at ~ 36 months	cm	NA		2,514	95.88 (3.79)
Weight measures overall		796	9 (3) [5-10]	133,732	9 (4) [5-14]
Weight measures for ages	0–12 months	280	3 (1) [3-5]	86,705	6 (0) [4-8]
	13–24	267	3 (1) [1-4]	31,809	3 (2) [0–4]
	25–36	249	3 (1) [0–5]	15,218	1 (2) [0–4]
Height measures overall		NA		107,586	10 (3) [5–13]
Height measures for ages	0–12 months	NA		68,927	6 (1) [3–8]
	13–24			26,221	3 (1) [0–4]
	25–36			12,438	1 (2) [0–3]
Ethnicity	Hispanic/Latino	62	63.9	1,026	6.9
	Non-Hispanic	35	36.1	8,418	56.8
	Unspecified	0		5,373	36.3
Race group	Asian	16	16.5	3,220	21.7
	Black	5	5.2	255	1.7
	Native American	0		18	< 1
	Pacific Islander	2	2.1	42	< 1
	White	73	75.3	3,911	26.4
	Other	1	1.0	1,858	12.5
	Unspecified	0		5,513	37.2

^a^Percent *or* mean (standard deviation [sd]) *or* median (interquartile range [IQR]) [range]

^b^ *NA* Not applicable in STORK (neither birth length nor height values were ascertained at household visits)

^c^ ± 2 months

### Weight models

The Michaelis–Menten model was successfully fitted to 94 STORK babies (95.9%) and 14,596 STARR babies (99.3%). The c1 parameter followed a normal distribution and approximated birthweight (Spearman Rho correlation: 0.79, 0.84 and 0.87 for STORK boys, STORK girls and both STARR boys and girls, respectively; difference between mean c1 values and mean birth weight: 0.30, 0.14, 0.06 and 0.05 kg in STORK boys, STORK girls, STARR boys and STARR girls, respectively) (Table 2, Supplemental Fig. 2). Distributions of the model parameters a1 and b1 were right-skewed; extremely high a1 and b1 parameters indicated linear growth, and a higher b1 to a1 ratio indicated both less rapid early growth in the infants and a more linear curve overall. The parameter values for a1 and b1 were weakly correlated with the c1 parameter value, indicating that birth weight might play a role in predicting these values (Spearman's Rho correlation ~ 0.30). Apart from the shape of the growth curve and the location of the inflexion point, however, we did not discern a physiological meaning for either a1 or b1.

**Table 2:** Weight and height modeling: Distribution of parameters a1, b1, c1 and birth weight or length for STORK and STARR infants, by sex, with goodness of fit (RMSE)

		**STORK** (*N* = 93)				**STARR** (*N* = 14,596 with weights, *N* = 11,626 with heights)			
**WEIGHT**		**a1**	**b1**	**c1**	**BW (kg)**	**a1**	**b1**	**c1**	**BW (kg)**
***Boys***	Mean	18.9	885	3.80	3.50	16.0	531	3.40	3.34
	sd	11.0	1,175	0.49	0.41	16.3	1,104	0.58	0.50
	Median	15.5	567	3.72	3.45	14.1	393	3.40	3.35
	IQR	6.89	450	0.67	0.62	5.77	327	0.75	0.63
	Range	9.84—72.2	151 – 7,964	2.82—4.81	2.73—4.38	4.34—709	73.1 -56,713	1.04—5.90	1.11—5.41
	RMSE (kg) Mean (sd)	0.475 (0.177) 0.467 (0.201) 0.647				0.245 (0.139) 0.222 (0.187) 0.431			
	Median (IQR)								
	90% <								
***Girls***	Mean	34.6	1,608	3.48	3.34	18.1	741	3.28	3.23
	sd	108	5,578	0.57	0.48	47.6	2,962	0.54	0.48
	Median	16.3	707	3.42	3.39	14.7	499	3.28	3.24
	IQR	9.52	540	0.66	0.56	6.77	425	0.68	0.61
	Range	7.99—746	117 – 3,8407	1.87—4.87	1.97—4.58	4.53—3,330	33.5—199,562	0.92—6.02	1.10—5.95
	RMSE (kg) Mean (sd)	0.459 (0.221) 0.434 (0.324) 0.737				0.221 (0.130) 0.198 (0.171) 0.395			
	Median (IQR)								
	90% <								
**HEIGHT**^a^						**a1**	**b1**	**c1**	**BL (m)**
***Boys***	Mean					61.4	469	51.1	50.8
	sd					15.8	243	2.50	2.58
	Median					62.0	502	51.0	50.6
	IQR					17.7	266	3.23	3.05
	Range					23.6—349	53.7 – 4,761	38.6—59.0	38.1—58.4
	RMSE (cm) Mean (sd)					0.962 (0.388) 0.932 (0.532) 0.998			
	Median (IQR)								
	90% <								
***Girls***	Mean					66.1	596	50.3	49.9
	sd					27.2	403	2.40	2.52
	Median					64.5	547	50.5	50.0
	IQR					19.6	320	3.03	3.05
	Range					22.7—882	54.0 – 12,557	38.9—57.8	38.6—58.4
	RMSE (cm) Mean (sd)					0.933 (0.373) 0.910 (0.495) 0.998			
	Median (IQR)								
	90% <								

Parameters for a1 and b1 are not normally distributed, so median and IQR values are more appropriate. Birth weights/heights and c1 parameters are normally distributed, so mean and standard deviations values are appropriate. All values are shown for sake of completeness

Study *N* = : total subjects who fit the model without error

*BW* Birthweight, *BL* Birth length, *IQR* Interquartile range, *RMSE* Root mean squared error, *sd* Standard deviation

90% < : 90% of subjects with RMSE less than

^a^Height information was not available for STORK babies

**Fig. 1:**
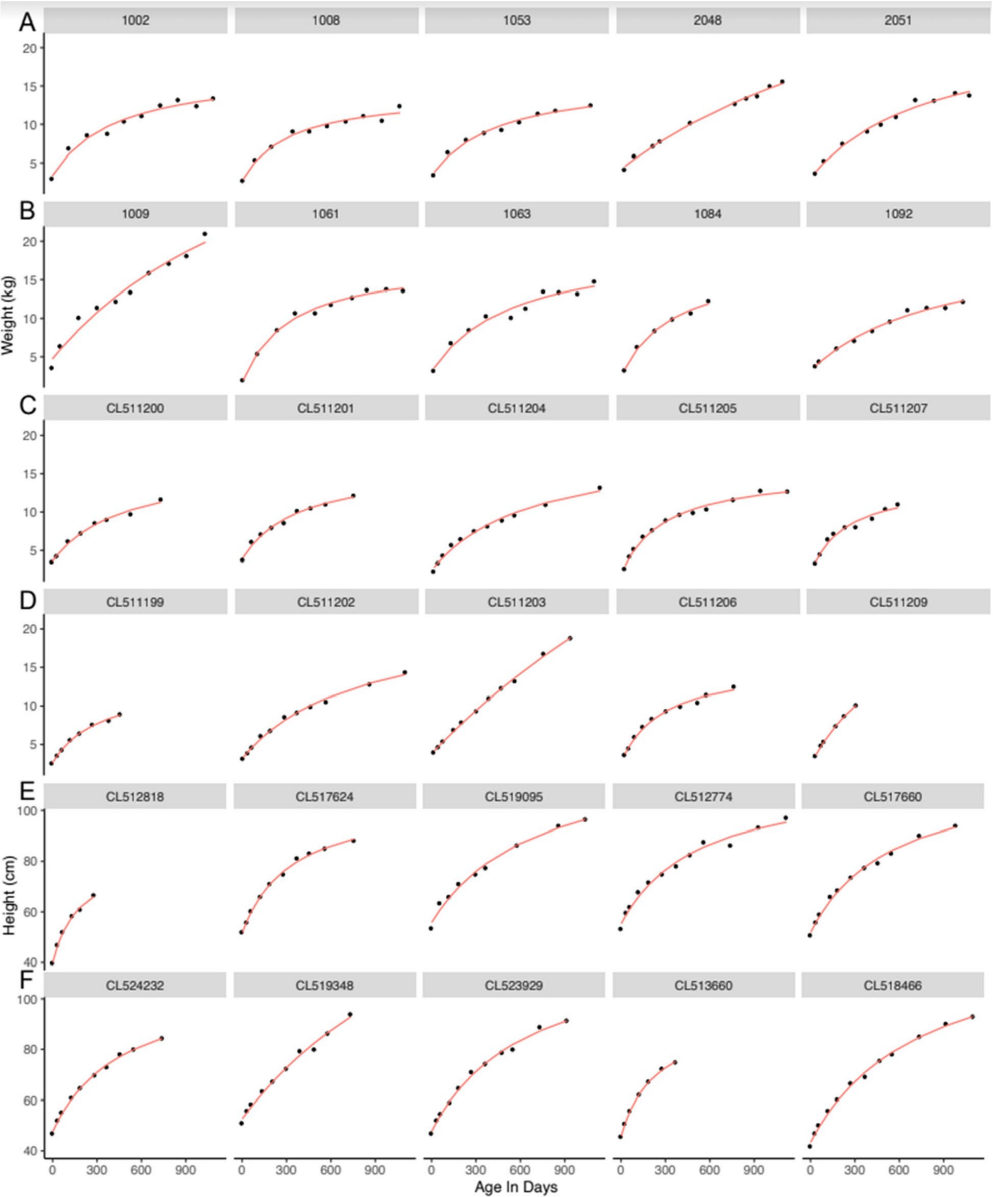
**A-F** A representative sample of fitted models for weight (kg) and for height (cm). Weight fitting (in kg) shown for: (**A**) STORK boys, (**B**) STORK girls, (**C**), STARR boys, (**D**) STARR girls, and height fitting (in cm) for: (**E**) STARR boys, (**F**) STARR girls. Each row shows the first five individuals from each given category in the dataset. The red line indicates the fitted model, and the black circles indicate actual weights or heights

**Fig. 2:**
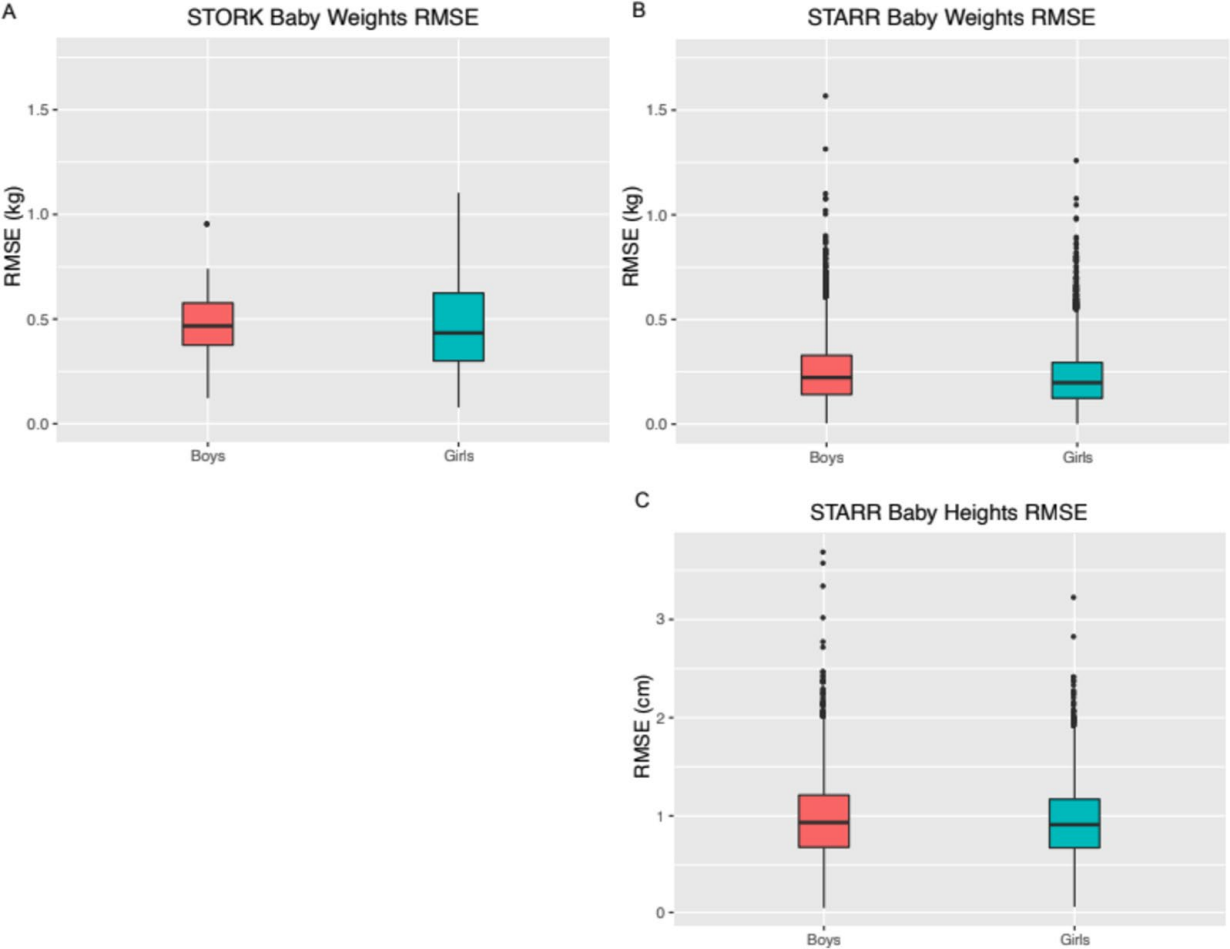
**A-C** Distribution of RMSE values for the modified Michaelis–Menten equation in babies by sex for weight (kg) and for height (cm). (**A**) STORK weights, (**B**) STARR weights, and (**C**) STARR heights

Visual inspection of plots of infant weights over time indicated a good fit with this model for all babies (Fig. 1, A-D). Model fit was high, as measured by low RMSE (Fig. 2A-B, Table 2). Overall, only 11 (0.08%) babies had RMSE values above 1.0 kg (Supplemental Fig. 3). The different ethnic/racial groups had similar RMSE values (Table 1, Supplemental Fig. 4). The effect of age on RMSE over time showed a slight increase across three years (Supplemental Fig. 5).

The model failed to fit 4.1% of STORK babies and 0.7% of STARR babies; these tended to show linear (vs. non-linear) growth (Supplemental Fig. 6).

### Height models

The model parameters a1 values were slightly left-skewed whereas the b1 values were right-skewed, with both showing a small number of large outliers; the c1 parameter again had a normal distribution and was correlated with birth length (Spearman Rho: 0.92 and 0.91 for boys and girls, respectively; difference between mean c1 value and mean birth length: 0.3 cm and 0.4 cm for boys and girls, respectively) (Table 2, Supplemental Fig. 7).

Visual inspection of the fitted data for height indicated excellent model fit (Fig. 1, E–F) and RMSE values were low (Fig. 2C), with both median and 90% values under 1 cm. Only five subjects (0.043%) had RMSE over 3 cm (Supplemental Fig. 8). RMSE values were similar across racial/ethnic groups (Supplemental Fig. 4). Similar to weight models, RMSE increased very slightly across time (Supplemental Fig. 5).

Very few babies (0.3%) failed to fit the model as a1 and b1 parameters increased without bound, showing either very linear growth or very large height values (Supplemental Fig. 9).

### Imputation tests

Considering growth only in the first year, the removal of visit1 (birth weight or length) increased RMSE more than the removal of any other recommended well-baby visit (Supplemental Table 1); the visit at approximately 12 months had the second largest impact on model fit. Considering growth over three years, while removal of birth weight had a large impact on RMSE, removal of any other individual well-baby visit alone had a far more modest effect. Many combinations of up to three visits in year 1 and up to five visits in years 1–3 could be dropped with only a small increase in RMSE, leaving as few as four visit timepoints needed in year 1, and as few as seven visit timepoints needed in years 1–3, with exceptions: removal of combinations of visit1 with other visits, particularly during year 1, led to a sizable increase in RMSE, as did removal of consecutive visits at the final time points (visits 5–7 for the year 1 subset; visits 10–12 for the years 1–3 subset). The RMSE could be rescued partly for missing visit1 data by increasing the initial a1 and b1 parameters to higher values (e.g., a1 = 15, b1 = 500).

### Prediction

Sufficient data for weight prediction modeling was available for 4,829 STARR infants (Supplemental Fig. 1); of these, 1.8% were dropped due to model failure to fit their growth curve. RMSE values for the full models with these babies were similar to models using all STARR babies. In modeling data from Y1 + Y2 to predict growth in Y3, RMSE increased by approximately 1.1 kg for weight and 2 cm for height, equivalent to 7.5% and 2.1% of sample mean weight and height at 36 months (Table 3; Supplemental Figs. 6 and 7; Table 1). Similarly, in modeling data from Y1 to predict growth in Y2 + Y3, RMSE increased to approximately 1.3 kg and 5.6 cm (8.8% and 5.8% of mean weight and height at 36 months, respectively).

**Table 3:** RMSE values for predicted weights and for predicted heights: Mean, median, IQR, and range for STARR predicted data

Model fit timepoints, RMSE timepoints	Mean	sd	Median	IQR	Range
**WEIGHT (kg)**					
***Boys***					
Y1-3, Y1-3	0.347	0.132	0.333	0.168	0.083–1.57
Y1-3, Y3	0.371	0.194	0.345	0.241	0.026–2.38
Y1-2, Y3	1.13	0.613	1.05	0.827	0.051–4.57
Y1-3, Y2-3	0.352	0.150	0.333	0.177	0.073–1.91
Y1, Y2-3	1.37	0.765	1.25	0.976	0.107–6.89
***Girls***					
Y1-3, Y1-3	0.312	0.123	0.296	0.160	0.058–1.08
Y1-3, Y3	0.340	0.182	0.313	0.225	0.025–1.79
Y1-2, Y3	1.08	0.187	1.03	0.840	0.046–4.17
Y1-3, Y2-3	0.319	0.623	0.298	0.168	0.045–1.45
Y1, Y2-3	1.34	0.810	1.19	1.04	0.131–6.63
**HEIGHT (cm)**					
***Boys***					
Y1-3, Y1-3	1.16	0.342	1.14	0.455	0.259–3.58
Y1-3, Y3	1.12	0.533	1.05	0.690	0.068–5.42
Y1-2, Y3	3.16	1.71	2.91	2.52	0.222–9.61
Y1-3, Y2-3	1.13	0.394	1.09	0.506	0.155–3.87
Y1, Y2-3	5.57	2.82	5.38	4.28	0.518–21.2
***Girls***					
Y1-3, Y1-3	1.11	0.315	1.08	0.429	0.326–2.42
Y1-3, Y3	1.06	0.494	0.991	0.647	0.085–3.12
Y1-2, Y3	2.94	1.64	2.68	2.22	0.133–11.8
Y1-3, Y2-3	1.07	0.360	1.04	0.456	0.187–2.42
Y1, Y2-3	5.76	2.99	5.64	4.56	0.447–21.0

The RMSE for model fitted to all years of data, as well as the RMSE calculated for the time window subsets are shown (i.e. the model is fit to the full data, but the RMSE is only calculated with the predicted values versus true values for Y2-3 or Y3)

*IQR* Interquartile range, *RMSE* Root mean squared error, *sd* Standard deviation

### Comparison with other models

Using weight holdout testing, RMSE values were comparable between the modified Michaelis–Menten equation and three of the ten models (Wand, SITAR and FACE; mean RMSE ~ 0.3 kg for all four models) with the remaining models showing higher RMSE values (Supplemental Table 2). Using height holdout testing, RMSE values were lowest for the modified Michaelis–Menten equation, slightly higher for the FACE and SITAR models and substantially higher for the remaining eight models (Supplemental Table 2).

## Discussion

Using longitudinal weight data first in a small birth cohort and subsequently in a large healthcare database, we found that a modified Michaelis–Menten equation described individual babies’ non-linear growth in weight and height from birth to age 36 months with minimal error. Although certain time points were essential for best model fit (birth weight or length, and, for year 1 growth, the measure at approximately 12 months), the loss of most other data points had only modest effects on RMSE, indicating that our model can correctly interpolate weights and heights for the majority of infants, even when information from multiple well-baby visits is missing. When compared to ten models commonly used to interpolate or evaluate growth in pediatric populations, this equation was able to interpolate height better than all, and weight better than all but three which showed similar accuracy (Wand, FACE and SITAR models). Given routine baby follow-up, this equation provides an excellent method to estimate weight or height at any time point within the first three years of life, providing a useful tool for pediatric researchers interested in this timeframe.

The modified Michaelis–Menten equation has been shown previously to describe growth in a wide array of living organisms and in particular mammals, including primates [11]. We believe our study is the first to demonstrate its applicability in humans. This equation has the distinct advantage of being conceptually simple: although childhood height and weight are clearly influenced by a multitude of factors, normal growth over time with sufficient resources mirrors an elementary chemical reaction on consumable substrates. Although we believe this equation is likely generalizable to healthy babies in the USA, as no differences in growth patterns between healthy babies of different racial and ethnic backgrounds in our sample were observed, it remains to be determined whether this equation is valid for growth in premature babies, babies with severe illness or health conditions, babies in resource-poor environments, or for clinical suspicion of aberrant growth in an individual patient.

We examined how well the modified Michaelis–Menten equation predicted growth at 36 months and found that estimates based on data from ages 0–24 months were within approximately 2.1% of actual height and 7.5% of actual weight. This difference in precision between height and weight may be because height measurements are less subject to intrinsic variation than weight measurements [24]; additionally, height might be less prone to measurement error than weight, as children may be weighed with or without clothes. Using measures from only the first year of life to predict height and weight to 36 months was more imprecise (within 5.8% and 8.8% of actual height and weight, respectively). To date, we have found no models designed specifically to predict growth at three years of life; this equation may provide an interesting approach for identifying unexpectedly low or high growth within an individual child up to this age, without focusing on standardized growth curves. Of course, our model includes only the initial hyperbolic growth before age three years; different models should be used when considering other time frames when the growth rate changes significantly (i.e., at puberty).

Limitations of the Michaelis–Menten equation include failure of the model to fit growth in children with linear (vs. non-linear) growth; the proportion of such babies in our study, however, was small (~ 0.7% overall) and these babies could potentially be fit to a standard linear growth model. We were also unable to determine a physiologic interpretation for two of the three model parameters, although together they are important for shaping the growth curve. In this study, we limited our time frame from birth to 36 months; an evaluation of how far along the age spectrum this equation remains reliable would be of interest. It is important to note that body mass index (BMI), a function of height and weight, does not follow a similar curve. Finally, although weight and height have been considered useful measures of growth, growth trajectories—their derivatives—are perhaps of greater importance [25–27].

## Conclusions

A modified Michaelis–Menten equation is a useful tool to accurately describe weight and height in individual, racially and ethnically diverse infants aged 0–36 months in California. Whether this equation can similarly explain growth in premature babies, sick children in resource-poor environments and those in older age categories has yet to be evaluated. Growth over time in an individual baby, like that of many known organisms, mirrors the saturation curve of a basic enzymatic reaction.

### Supplementary Information

**Additional file 1. **

## Data Availability

The dataset and associated code supporting the conclusion of this article are available at datadryad.org (10.5061/dryad.4j0zpc8jf).
