# Supplementary material for: A modified Michaelis-Menten equation estimates growth from birth to 3 years in healthy babies in the USA
Source: BMC Med Res Methodol. 2024 Feb 1;24:27. doi: 10.1186/s12874-024-02145-1 (PMC10832211; doi:10.1186/s12874-024-02145-1)

Walters et al: A modified Michaelis-Menten equation estimates growth from birth to 3 years in healthy babies in the US.

SUPPLEMENTAL INFORMATION

LIST OF TABLES AND FIGURES

Supplemental Table 1:	RMSE and NLS failure when timepoints are removed, for STARR subject weights and heights.
Supplemental Table 2:	RMSE value of holdout datapoints for different interpolation methods and pediatric growth models.
Supplemental Fig 1:	Sample sizes for different analyses
Supplemental Fig 2:	Weight modeling: distribution of parameter values (STORK and STARR).
Supplemental Fig 3:	Weight modeling for all subjects with high RMSE values (>1kg)
Supplemental Fig 4:	Weight and height RMSE values by ethnicity and race.
Supplemental Fig 5:	RMSE versus age in days.
Supplemental Fig 6:	Weight over time values for all NLS fitting failures.
Supplemental Fig 7:	Height modeling: distribution of parameter values (STARR only).
Supplemental Fig 8:	Height modeling of all subjects with high RMSE values (>3.0cm).
Supplemental Fig 9:	Height over time values for all NLS fitting failures.
Supplemental Fig 10:	Weight prediction: Fitted curves for 10 randomly selected children (50% boys) using models fit from early time frames (STARR only).
Supplemental Fig 11:	Height prediction: Fitted curves for 10 randomly selected children (50% boys) using models fit from early time frames (STARR only).

OTHER INFORMATION

See Dryad.com for source data and the associated R code.

An R script to run the modified Michaelis-Menten equation can be downloaded at: https://gist.github.com/walterst/ede8b883d4f9acaf45ec9e2b0ec811fe.

Supplemental Table 1: RMSE and NLS failure when timepoints are removed, for STARR subject weights and heights.

This is tested for year 1 data (visits 1-7) and year 1-3 data (visits 1-12); from 1 to 3 visits are dropped in year 1 and from 1-5 visits are dropped in years 1-3. Date ranges for the visits are indicated in the table. “fulldata” indicates that no visits were dropped, and is the baseline in the RMSE imputation. Numbers after the visit indicate which visit(s) were removed, and the RMSE statistics under each visit reflect the impact on accuracy of the imputations due to the missing visit(s).

See Supplemental_Table_1.xlsx

Supplemental Table 2: RMSE value of holdout datapoints* for different interpolation methods and pediatric growth models.

The hbgd package from Anderson et al. was used for modeling STARR data other than for: (1) the 'last observation carried forward' model (the holdout data point was interpolated by the last observation by converting the random holdout value to NA and then using the function na.locf() from the zoo R package); (2) the simple linear model (the holdout-filtered data were used to determine the slope and intercept via R's lm() function, which were then used to calculate the holdout value); and (3) the SITAR model (each subject was fitted calling the sitar() function with df=2 to minimize failures, and the RMSE of the random holdout point was subsequently calculated with the predict() function).

**Method**	**Mean RMSE[a]**	
	**Weight (Kg)**	**Height (cm)**
Modified Michaelis-Menten equation	0.307	1.209
Last observation carried forward	1.095	4.478
Linear model	0.791	3.162
Robust linear model (RLM)	1.339	4.010
Laird and Ware linear model (LWMOD)	0.709	2.107
Generalized additive model (GAM)	0.449	2.339
Locally estimated scatterplot smoothing (LOESS)	0.939	3.385
Cubic smoothing spline (smoothspline)	0.469	2.473
Multilevel spline model (Wand)	0.324	6.284
Superimposition by translation and rotation. (SITAR)	0.333	1.336
Fast covariance estimation (FACE)	0.305	1.323

[a] The modified Michaelis Menten equation did not perform well for predicting birth weight or height (see Supplemental Table 1) so these datapoints were not included as potential holdout values.

Supplemental Figure 1: Sample sizes for different analyses

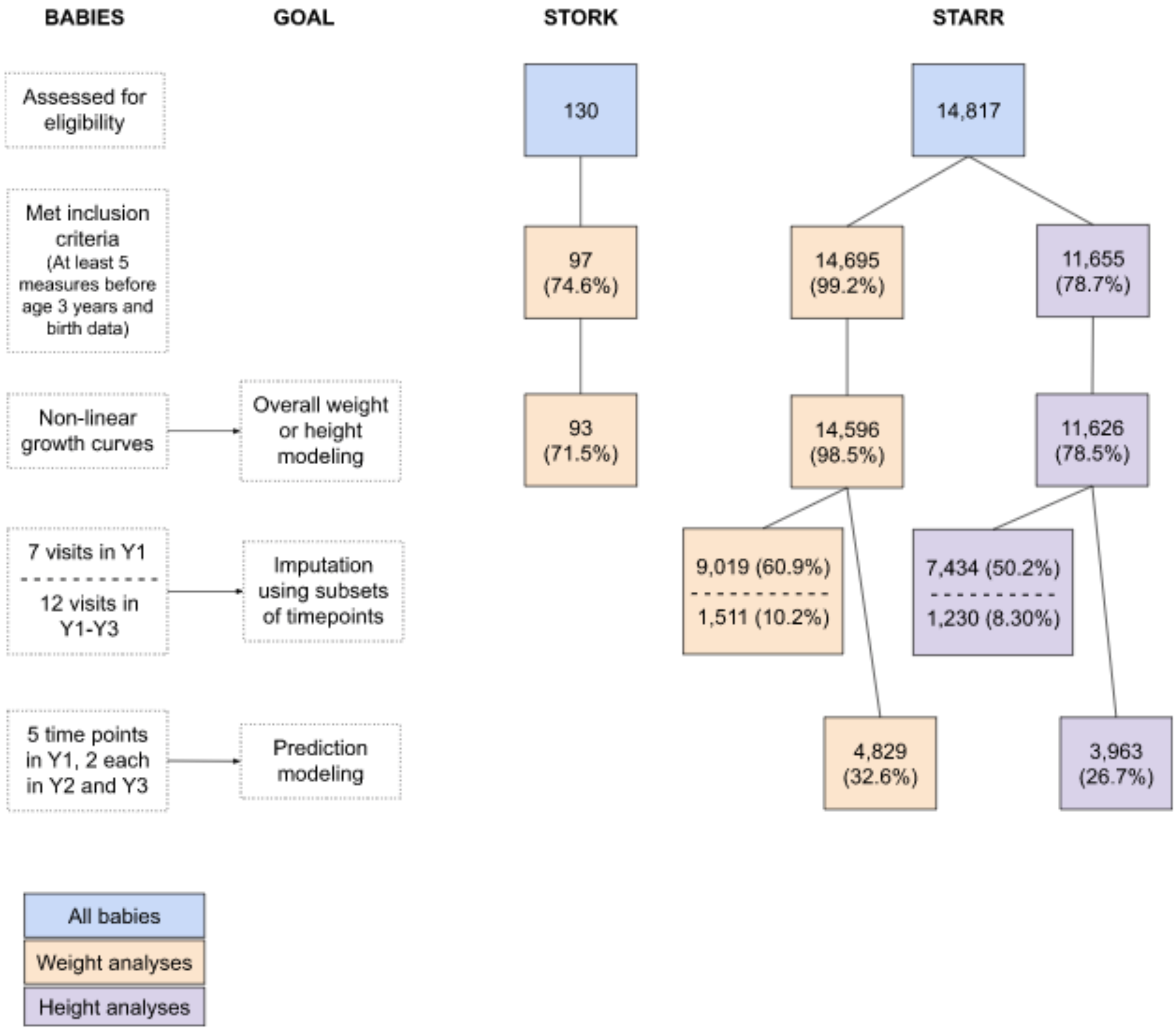

Supplemental Figure 2: Weight modeling: distribution of parameter values (STORK and STARR). Histogram of weight parameter values, by STORK and STARR study and sex as calculated by the modified Michaelis-Menten NLS function. Columns indicate parameters: STORK female: A: a1, B: b1, and C. c1; STORK male: D: a1, E: b1, and F: c1; STARR female: G: a1, H: b1, and I: c1; and STARR male J: a1, K: b1, and L: c1. For a1 and b1 parameters, the bottom 95% of the data are shown.

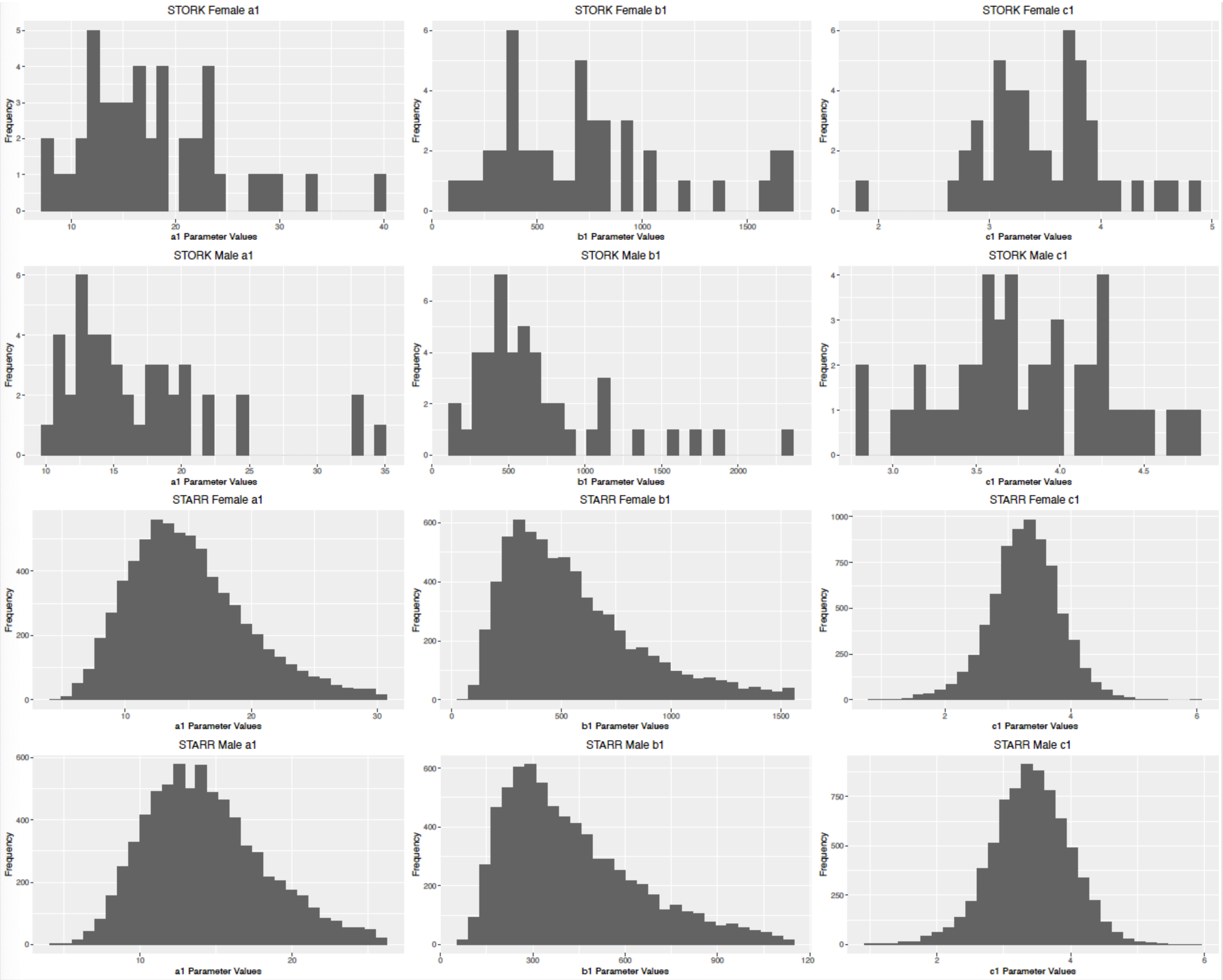

Supplemental Figure 3: Weight modeling for all subjects with high RMSE values (>1kg)
One STORK subject (2694) is shown; remaining subjects are from STARR. Black dots indicate the weight values and the fitted equation line is shown in red.

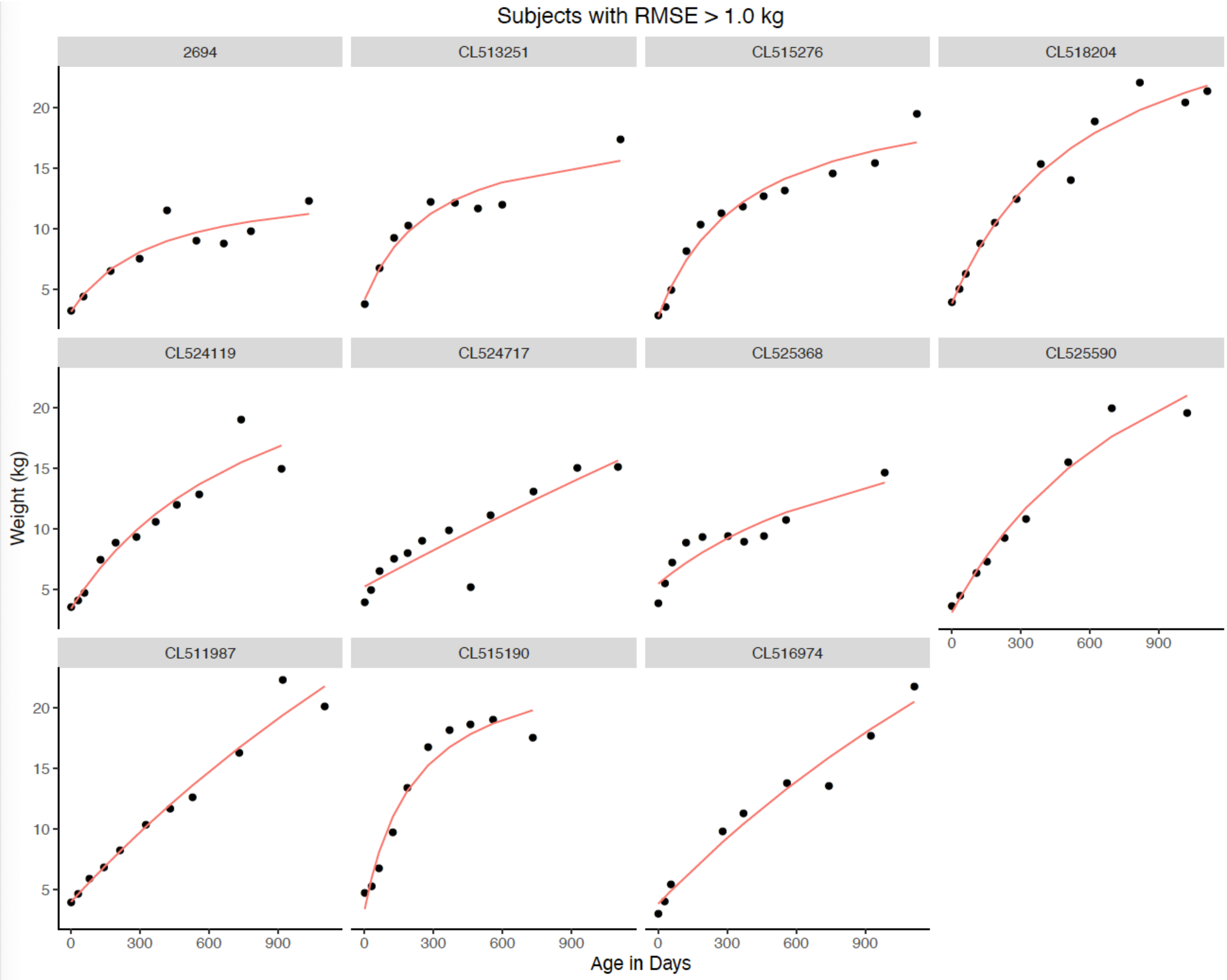

Supplemental Figure 4: **Weight and height RMSE values by ethnicity and race.** RMSE values for weight by (A) ethnicity and (B) race. RMSE values for height by (C) ethnicity and (D) race.

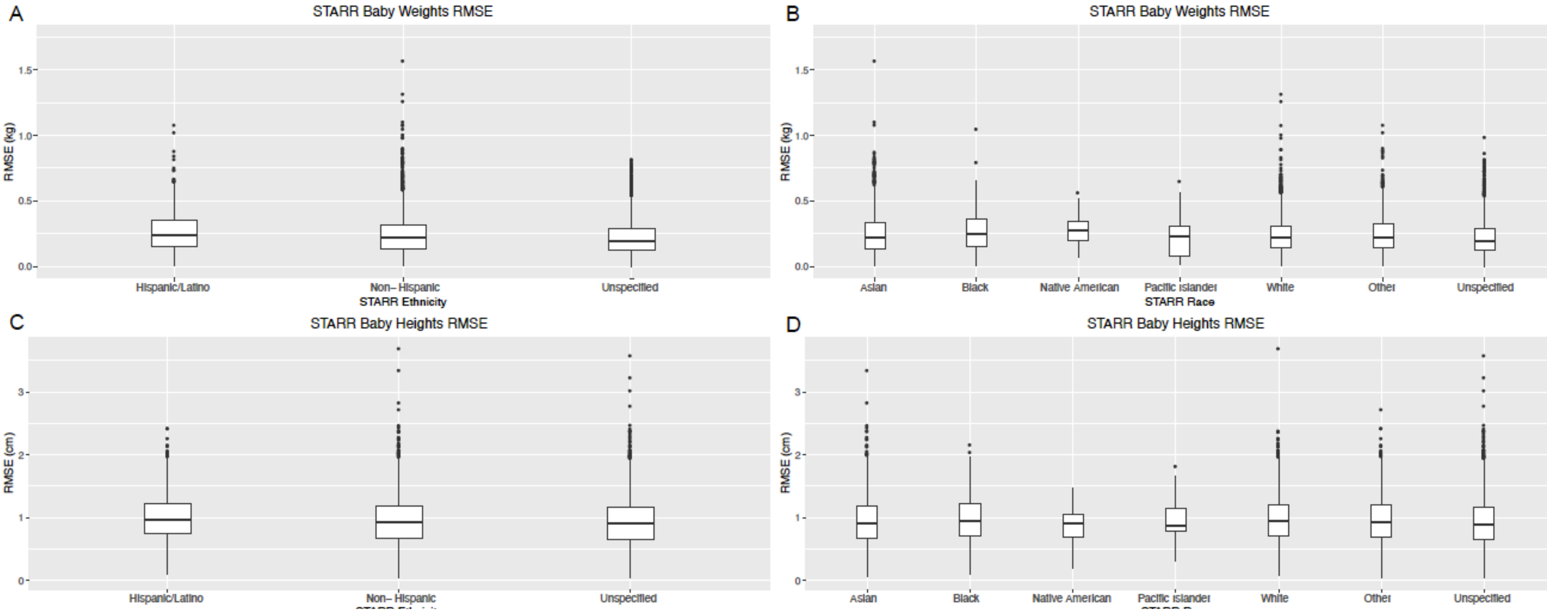

Supplemental Figure 5: RMSE versus age in days. A. RMSE of weight in kg vs age in days. B. RMSE of height in cm vs age in days.

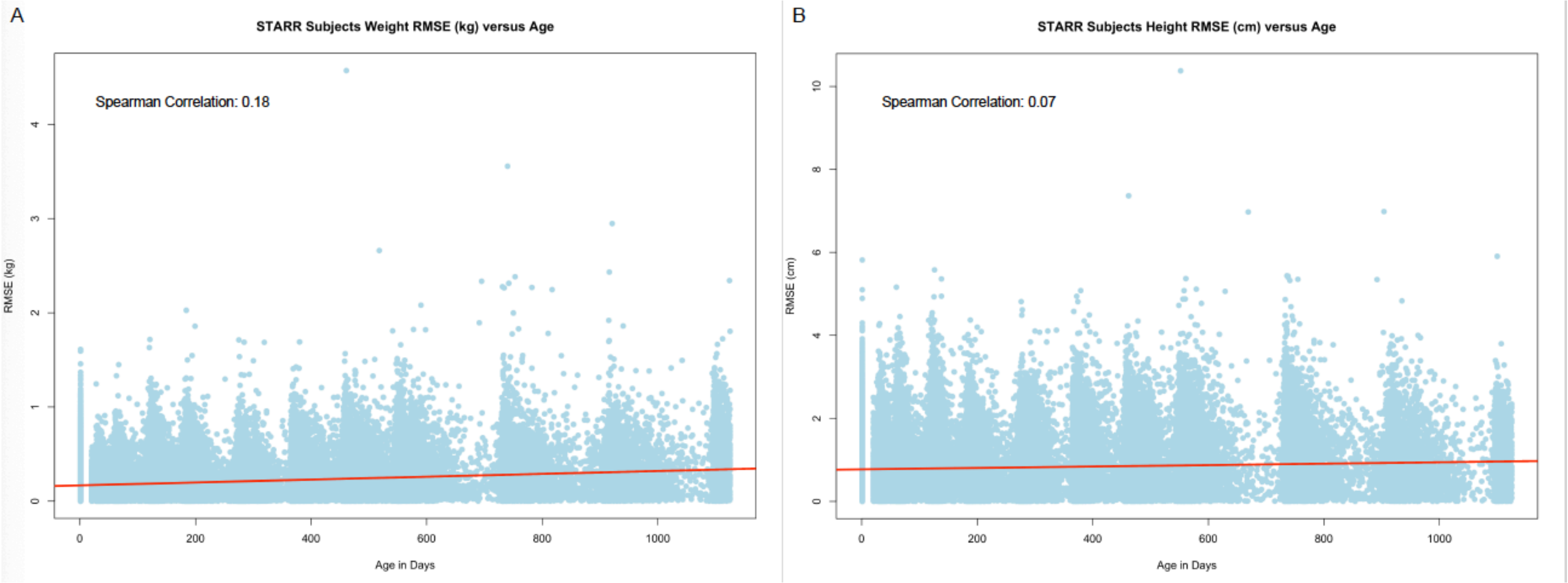

Supplemental Figure 6: Weight over time values for all NLS fitting failures. Weight over time data for STORK (first 4 IDs) and STARR subjects are shown for subjects that failed to return a non-linear fit with the nls() function.

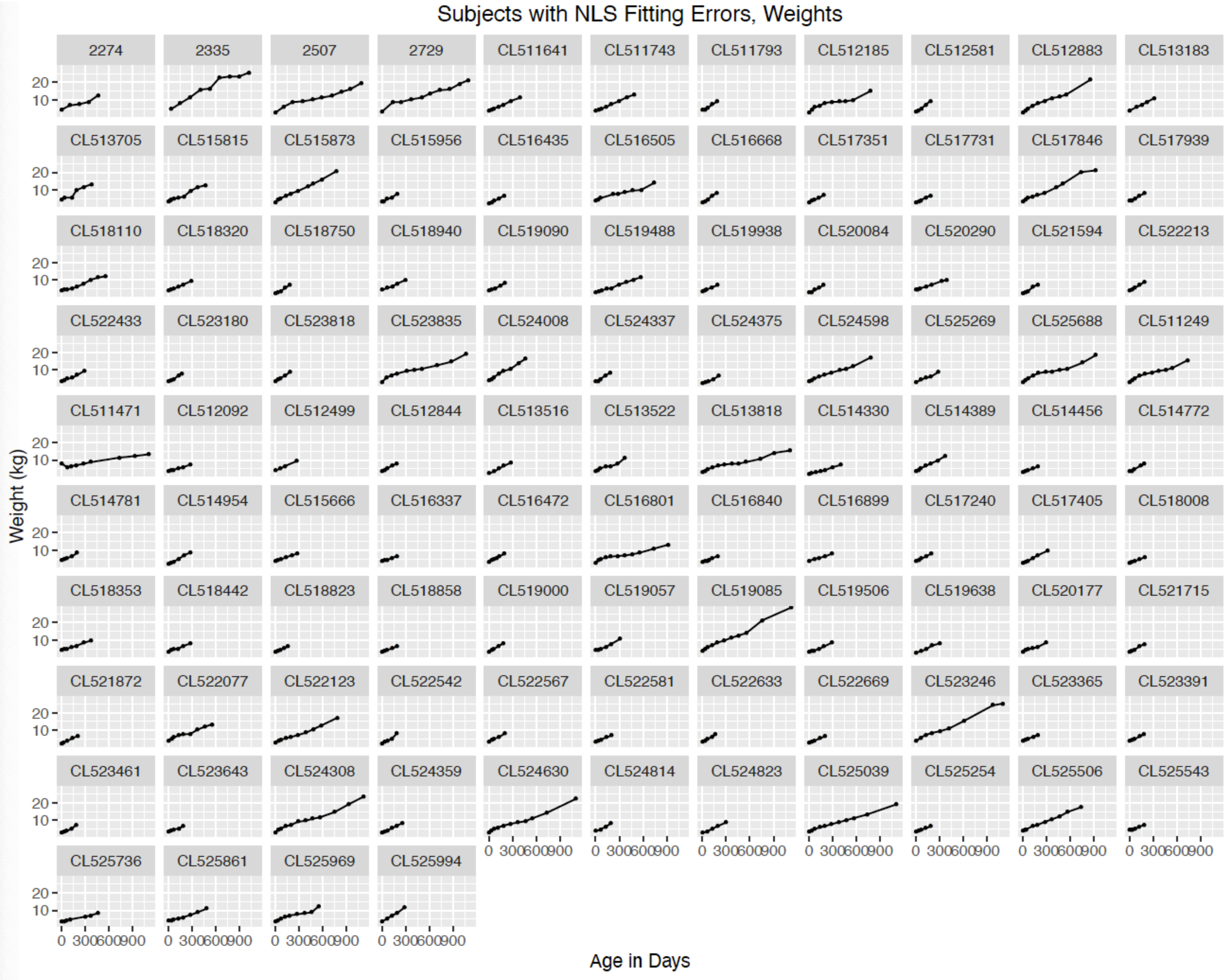

Supplemental Figure 7: **Height modeling: distribution of parameter values (STARR only)**. Histogram of height parameter values, by sex as calculated by the modified Michaelis-Menten NLS function. Columns indicate parameter: A: female a1, B: female b1, C. female c1, D: male a1, E: male b1 and F: male c1. For a1 and b1 parameters, the bottom 95% of the data are shown.

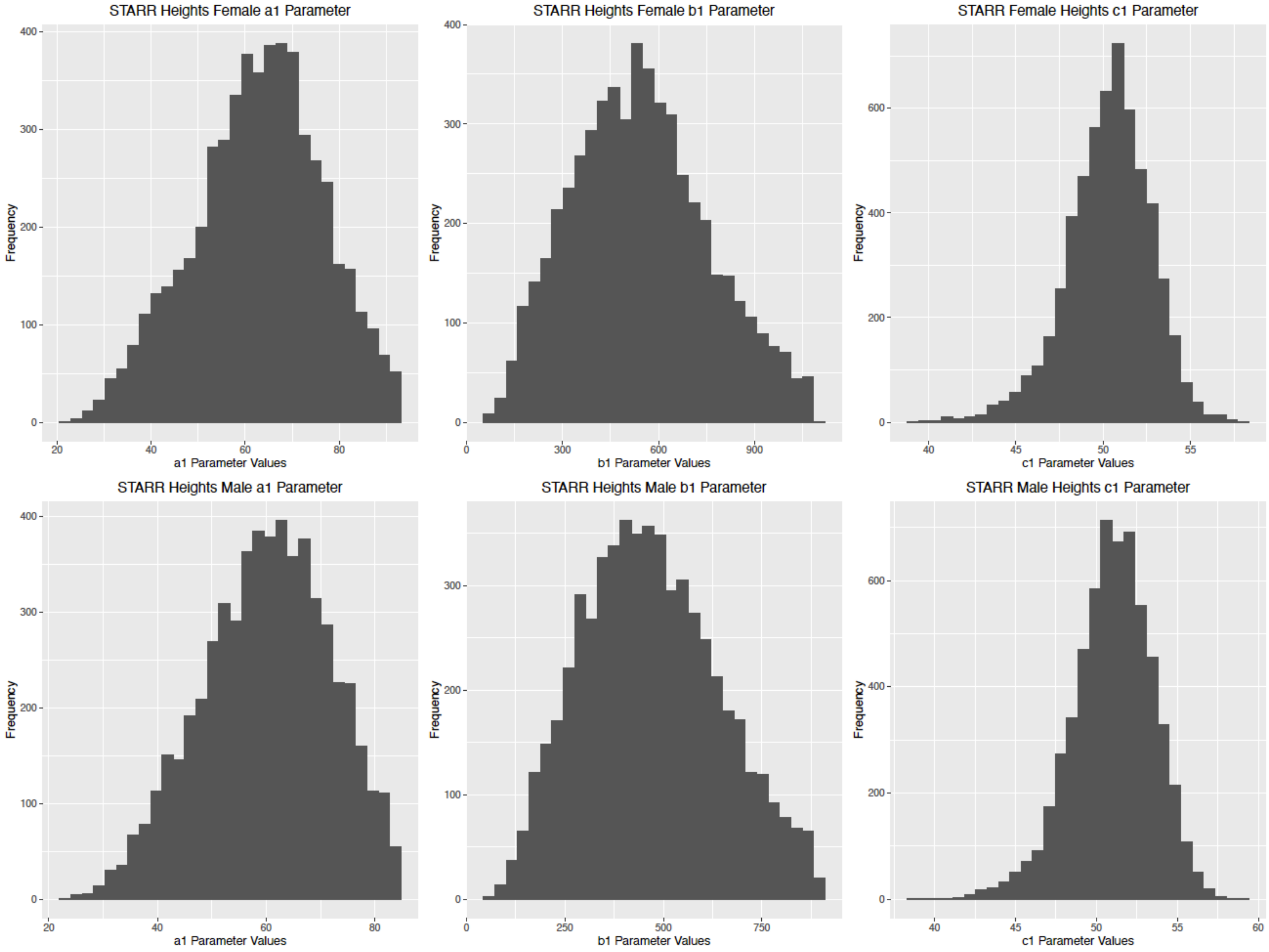

Supplemental Figure 8: Height modeling of all subjects with high RMSE values (>3.0cm). Black dots indicate the weight values and the fitted equation line is shown in red.

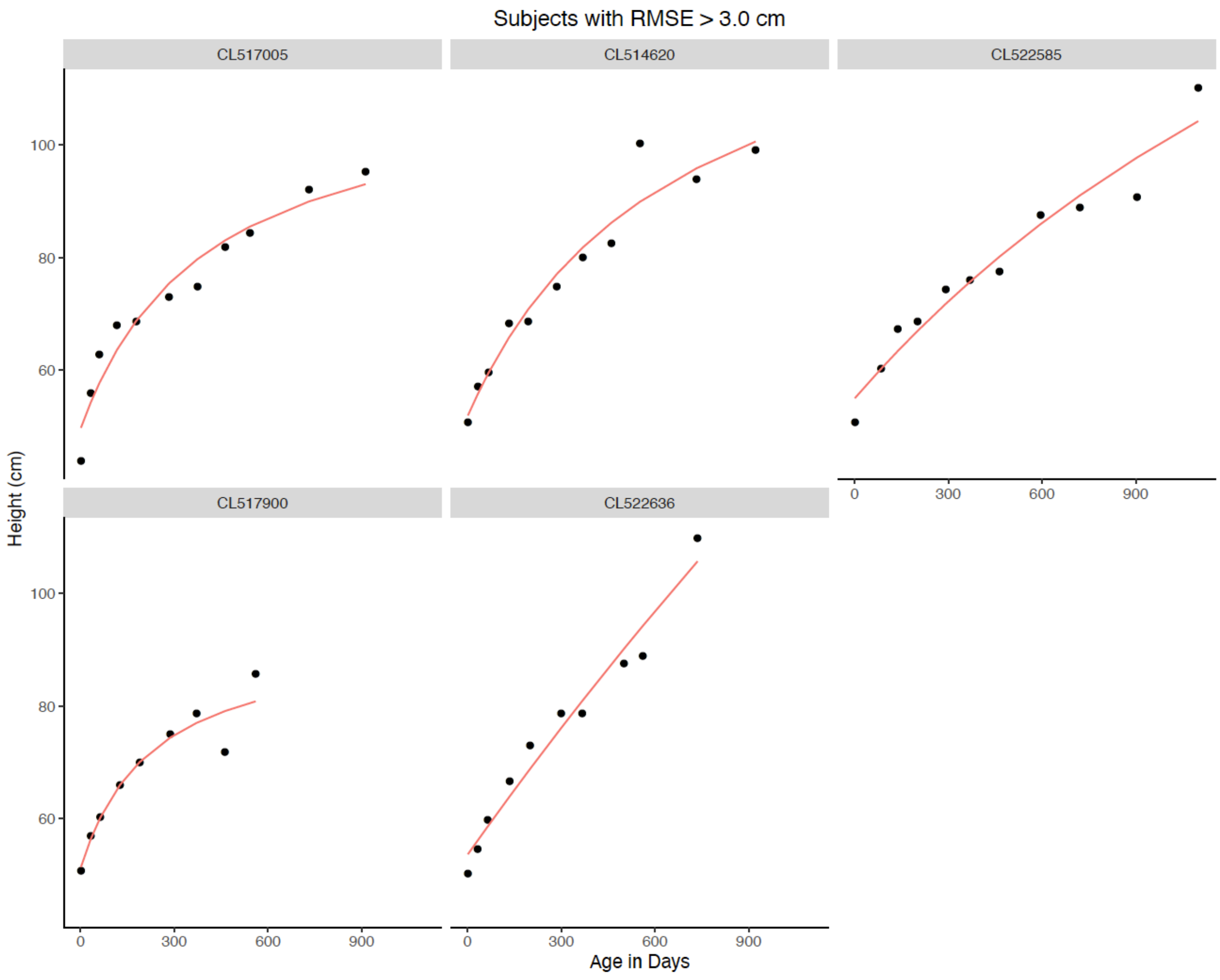

Supplemental Figure 9: Height over time values for NLS fitting failures. Height over time data for STARR subjects are shown for subjects that failed to return a non-linear fit with the nls() function.

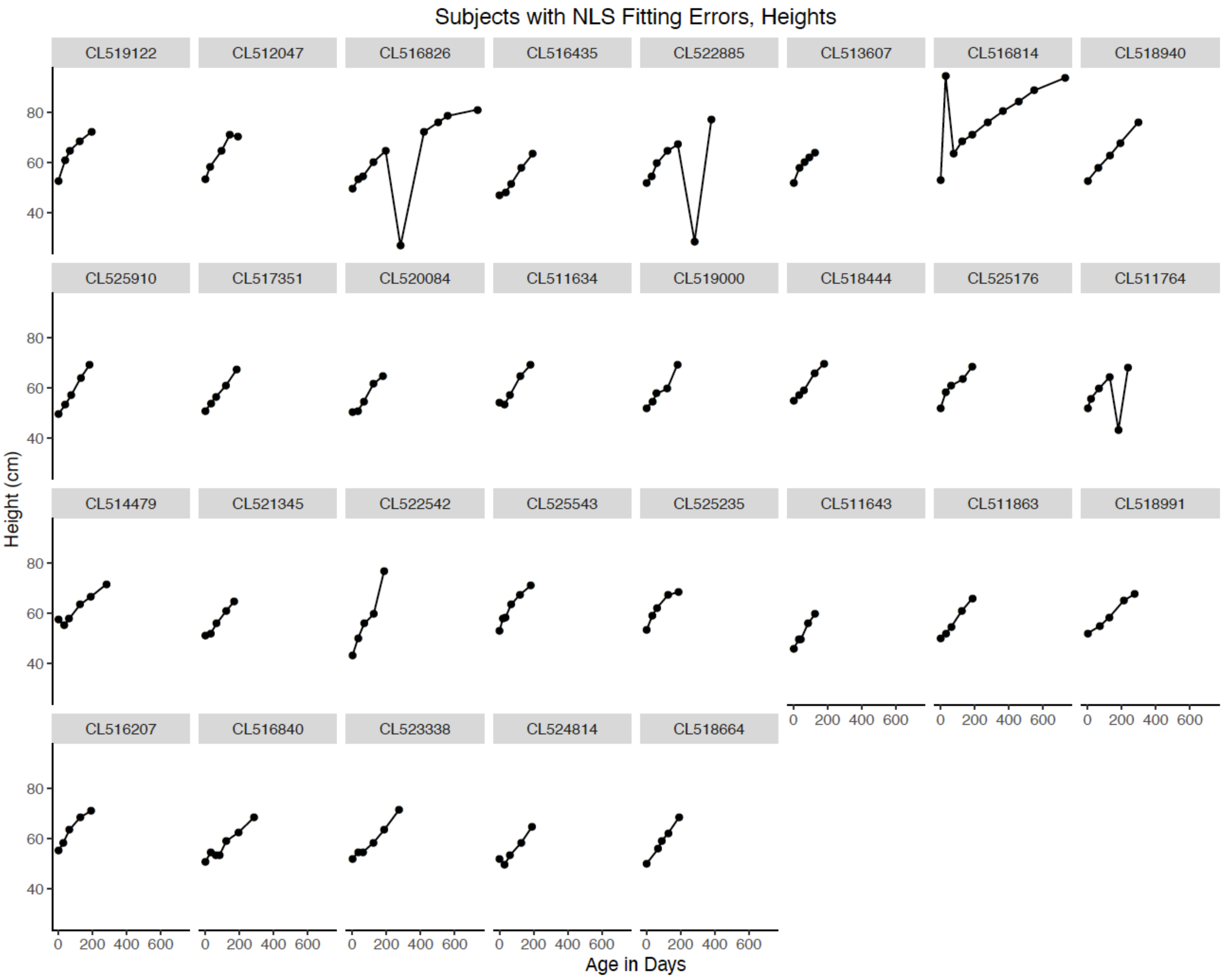

Supplemental Figure 10: Weight prediction: Fitted curves for 10 randomly selected children (50% boys) using models fit from early time frames (STARR only). Black dots indicate actual weights. Models were fit for the full data (black), years 1 and 2 (blue), and year 1 alone (orange).

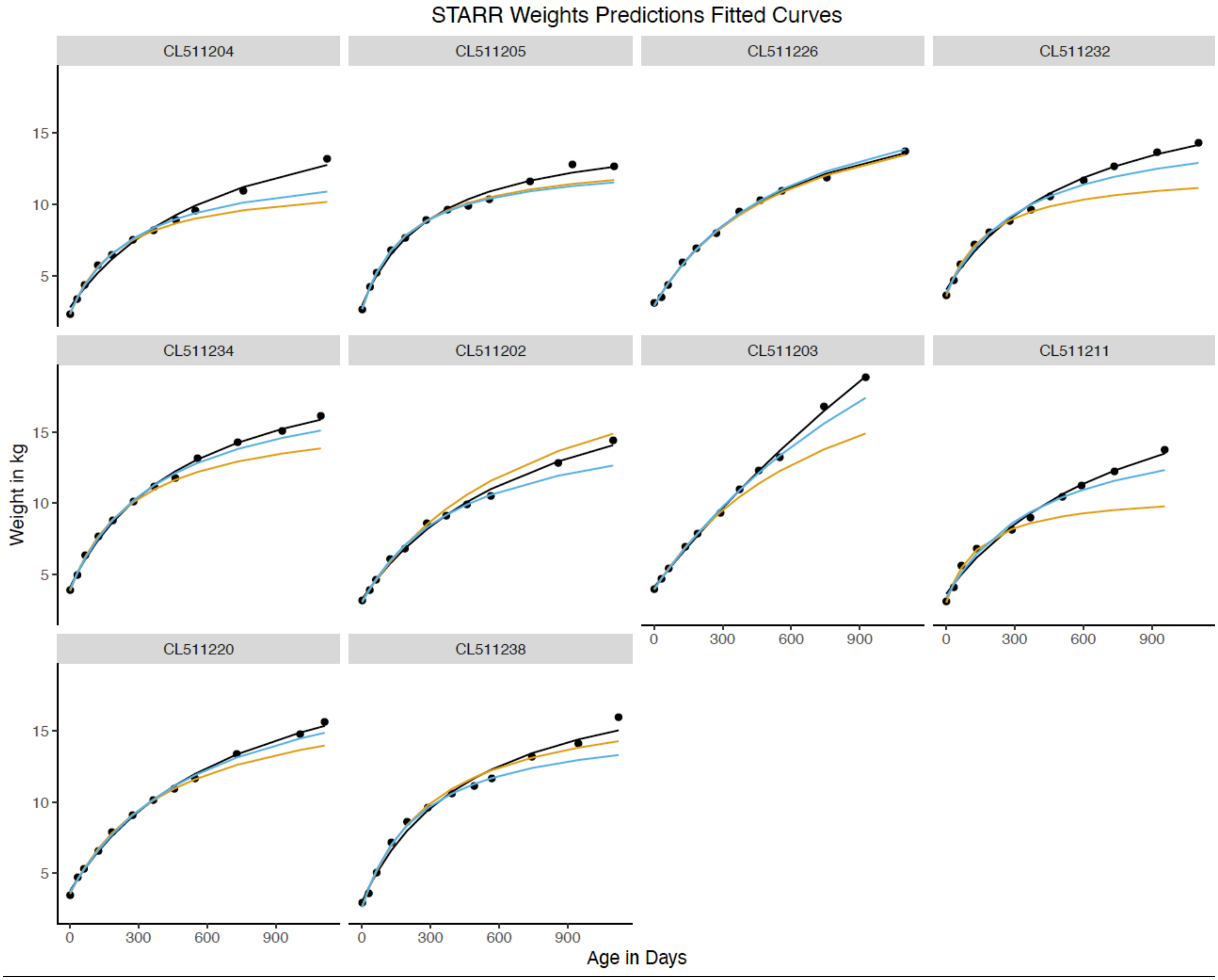

Supplemental Figure 11: **Height prediction: Fitted curves for 10 randomly selected children (50% boys) using models fit from early time frames (STARR only)**. Black dots indicate actual heights. Models were fit for the full data (black), years 1 and 2 (blue), and year 1 alone (orange).

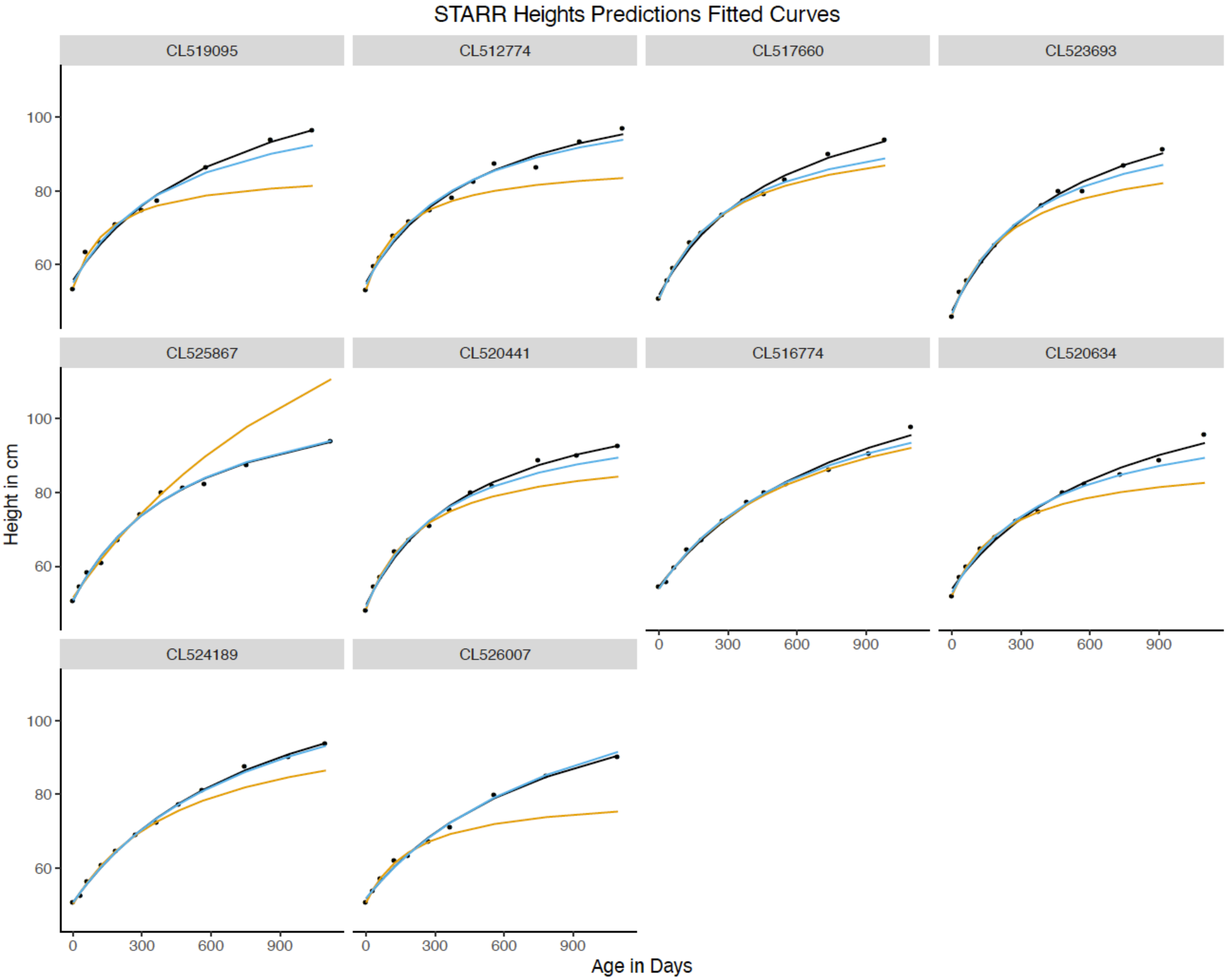

Supplement: Supplementary file 1 — Additional file 1. [file 12874_2024_2145_MOESM1_ESM.pdf]
